# Photoplethysmography based atrial fibrillation detection: a continually growing field

**DOI:** 10.1088/1361-6579/ad37ee

**Published:** 2024-04-17

**Authors:** Cheng Ding, Ran Xiao, Weijia Wang, Elizabeth Holdsworth, Xiao Hu

**Affiliations:** 1 Nell Hodgson Woodruff School of Nursing, Emory University, Atlanta, GA, United States of America; 2 The Wallace H. Coulter Department of Biomedical Engineering, Georgia Institute of Technology, Atlanta, GA, United States of America; 3 Georgia Tech Library, Georgia Institute of Technology, Atlanta, GA, United States of America; 4 Department of Biomedical Informatics, Emory University School of Medicine, Atlanta, GA, United States of America

**Keywords:** photoplethysmography, atrial fibrillation, statistic, machine learning, deep learning

## Abstract

*Objective.* Atrial fibrillation (AF) is a prevalent cardiac arrhythmia associated with significant health ramifications, including an elevated susceptibility to ischemic stroke, heart disease, and heightened mortality. Photoplethysmography (PPG) has emerged as a promising technology for continuous AF monitoring for its cost-effectiveness and widespread integration into wearable devices. Our team previously conducted an exhaustive review on PPG-based AF detection before June 2019. However, since then, more advanced technologies have emerged in this field. *Approach.* This paper offers a comprehensive review of the latest advancements in PPG-based AF detection, utilizing digital health and artificial intelligence (AI) solutions, within the timeframe spanning from July 2019 to December 2022. Through extensive exploration of scientific databases, we have identified 57 pertinent studies. *Significance.* Our comprehensive review encompasses an in-depth assessment of the statistical methodologies, traditional machine learning techniques, and deep learning approaches employed in these studies. In addition, we address the challenges encountered in the domain of PPG-based AF detection. Furthermore, we maintain a dedicated website to curate the latest research in this area, with regular updates on a regular basis.

## 1. Introduction

AF is a highly prevalent cardiac arrhythmia, which affects approximately 1%–2% of the general population, and is expected to continue to rise in the future worldwide due to population aging (Schnabel *et al* 2015, Lane *et al* 2017, Vinter *et al* 2020). Individuals with AF face a substantially heightened risk of experiencing cerebral and cardiovascular complications. Specifically, they are at a five fold higher risk (Tsao *et al* 2022) of ischemic stroke and are associated with an increased risk of ischemic heart disease, sudden cardiac death, and heart failure (Odutayo *et al* 2016). In general, people with AF have a four times increased risk of mortality compared to the general population (Lee *et al* 2018). The current detection of AF heavily relies on routine medical examinations; however, this approach may overlook paroxysmal AF cases, which refer to AF episodes that occur sporadically and self-terminate within 7 d. Additionally, a significant portion of AF patients, estimated at 25%–35%, remain asymptomatic (Rienstra *et al* 2012), which further reduces their likelihood of seeking care. These factors collectively contribute to delays in the identification of AF cases. Consequently, there has been a surge in efforts from both industry and academia sectors for developing technologies that enable reliable and continuous detection of AF. These advancements aim to transform the screening process for early detection of AF, particularly by identifying asymptomatic cases, potentially altering the course of treatment, and necessitating further research to fully understand their impact on patient outcomes (Boriani *et al* 2014, Chen *et al* 2018).

To enable consistent and long-term monitoring of atrial fibrillation (AF), a solution needs to be non-intrusive, cost-effective, and convenient, reducing operational complexity and encouraging user compliance. To this end, photoplethysmography (PPG) has emerged as a preferred technology, with a ubiquitous adoption in over 71% of wearable devices given its capacity to capture heart rhythm dynamics (Charlton *et al* 2023). The physiological foundation of PPG for AF detection lies in the fact that irregular heartbeats induce variations in cardiac output, leading to fluctuations in peripheral blood volume. This results in irregular pulse-to-pulse intervals and altered morphologies in PPG during AF episodes. Exploiting this physiological basis, wearables equipped with PPG sensors and specialized software offer great promise for personalized self-monitoring of AF, enabling individuals to receive timely alerts for potential AF episodes. However, the success of this approach hinges on the accuracy of PPG AF detection algorithms. Suboptimal algorithms can easily lead to a surge in false positives, thereby straining healthcare resources through unnecessary or inappropriate medical consultations.

Therefore, it marks tremendous importance for the development of precise and sensitive PPG-based algorithms for AF detection. These algorithms should aim to minimize false detections and optimize the utilization of healthcare resources, ensuring that appropriate clinical guidance is provided to individuals experiencing actual AF episodes. A prior review conducted by Pereira *et al* provided a comprehensive summary of research on PPG-based AF detection using statistical analysis (STAT), machine learning (ML) and deep learning (DL) approaches up until July 2019 (Pereira *et al* 2020). The review concluded that PPG holds promise as a viable alternative to ECG for AF detection. However, it also highlighted challenges such as the presence of arrhythmias other than AF, motion artifacts in PPG signals from wearable devices, and labor-intensive data annotation processes, among others.

Given the rapid technological advancements in wearable technology and methodological development in artificial intelligence (AI), there is a well-justified need for an updated review of AF detection using PPG. Building upon the previous work by Pereira *et al*, this paper aims to fill the gap by providing a comprehensive review of the latest developments in utilizing PPG-based digital health and AI solutions for AF detection in both inpatient and outpatient settings from July 2019 to December 2022. The articles included in this review are classified by the three methodological categories established by Pereira *et al* (2020), namely, STAT, ML, and DL, to facilitate the tracking of evolving trends in the field. In addition to conducting a thorough analysis of studies on PPG-based AF detection, this study has established an online knowledge database (GitHub). This database encompasses all studies reviewed up to December 2022, including those from our work and Pereira’s, along with direct links to the respective papers. Committed to keeping the database current, our team will update it semi-annually. Through the creation of this resource, we aim to foster community collaboration and accelerate the development of effective solutions to this critical clinical challenge.

## 2. Search criteria

The research team used the SCOPUS, IEEE Xplore, PubMed, Web of Science, and Google Scholar databases to gather appropriate documents for the review. All articles selected were published between July 2019 and up to December 2022, and reviews were eschewed in favor of data-based research studies. Databases function similarly, but not uniformly, so queries needed to be adjusted to reflect this. Filters were used in all databases to restrict the date of publication. Table 1 describes the exact search strings used in different databases for initial document screening. After the documents were retrieved (in total 57 studies), they were further evaluated for appropriateness for review by two researchers (RX and CD). For the subsequent analysis, only studies focused on developing detection algorithms using PPG for AF detection were included. Review papers, perspectives, commentaries, clinical trials, and meta-analyzes were excluded from further analysis. Based on this search criteria, there are in total 57 studies included in the review, including 17 STAT, 18 ML, and 22 DL studies.

**Table 1.** Search strings used in different scientific databases for study screening.

Scientific database	Search strings
SCOPUS	(PPG or photoplethysmography) and (atrial fibrillation or AF or AFib or arrhythmia or cardiac rhythm) and (detection or recognition)
IEEE Xplore	(‘All Metadata’: atrial fibrillation) AND (‘All Metadata’: wearable computer) AND (‘All Metadata: photoplethysmography OR ‘All Metadata’: PPG)
PubMed	(PPG ‘OR’ Photoplethysmography) ‘AND’ (atrial fibrillation ‘OR’ AF ‘OR’ Afib ‘OR’ arrythmia of cardiac rhythm) ‘AND’ (detection ‘OR’ recognition)
Web of science	(PPG or Photoplethysmography)(All Fields) and (atrial fibrillation or AF or afib or arrythmia or cardiac rhythm)(All Fields) and (detection or recognition)(All Fields)
Google scholar	(PPG or Photoplethysmography) and (atrial fibrillation or AF or AFib or arrhythmia or cardiac rhythm) and (detection or recognition)

To categorize studies into STAT, ML and DL, the primary classifier adopted in the studies was considered as the determinant factor for characterization. This way, in mixed methods where, for instance, features traditionally belonging to ML are fed into a DL classifier, the overall assigned category would be considered as DL.

## 3. Publication trends in the past decade

**Figure 1.**
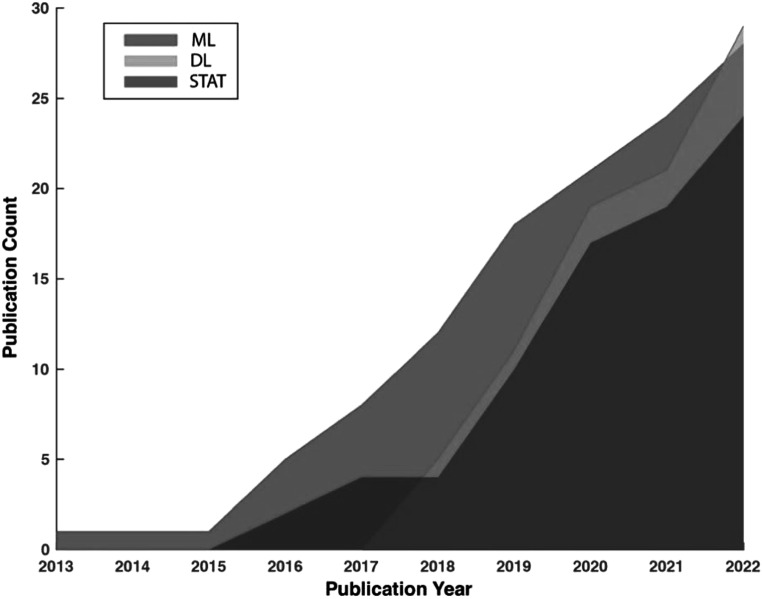
Trends in the cumulative numbers of publications in three method categories using PPG for AF detection.

Figure 1 depicts the trends in the cumulative number of publications in the three method categories in the past 10 years between January 2013 and December 2022. To maintain consistency, the same screening criteria were applied to identify relevant studies from before the review period of the current study. It reveals an accelerated rate of growth in the number of publications in all three categories, indicating the increasing effort outpouring to developing PPG-based AF detection algorithms. It is worth noting that studies utilizing DL for AF detection emerged in 2017 and expanded rapidly, outpacing the other two categories. In the year 2022, the cumulative number of publications using DL for AF detection exceeded any of the other two categories for the first time in history.

## 4. Review of recent studies on PPG-based AF detection

Tables 2–4 were adapted and extended based on previous work from Pereira *et al* (2020). These tables summarize the compiled studies for PPG-based AF detection categorized by three different signal processing methods. It is important to note that within the 57 studies reviewed, some studies employed more than one signal processing approach, leading to their inclusion in multiple tables, allowing for a comprehensive understanding of the various methodologies. More information on data train/test splitting and excluded data due to noisy signals or motion artifacts can be found in tables A1–A7 in appendix A for STAT, ML and DL studies, respectively.

**Table 2.** Studies on photoplethysmography based AF detection using statistical approaches.

Author (year) [Reference]	Number of patients	Dataset features	Age of population Mean (SD)	Length PPG segments	Measurement device	Acquisition conditions	Input data	Methodology	Performance results for rhythms detection
Väliaho *et al* (2019)	213	106 AF, 107 NSR	72.0 (14.3)	5 min	Wrist band	Outpatient-- checkpoint	Pulse-to-pulse interval	Two AF detection algorithms: AF Evidence and COSEn	Sen = 0.962; Spe = 0.981
Eerikäinen *et al* (2019)	32	13 continuous AF, 10 non-AF	AF: 70 (9) YO, Non-AF: 67 (13) YO	30 s	Data logger worn on the arm	Outpatient-- continuous measurement	Inter-pulse interval features: the percentage of inter- val differences of successive intervals greater than 70 ms (pNN70), Shannon Entropy (ShE), and Sample Entropy (SampEn)	Logistic regression	5 min data: Sen = 0.989; Spe = 0.990; Acc = 0.990; 24 h data: Sen = 0.970; Spe = 0.920; Acc = 93.91%
Kabutoya *et al* (2019)	59	29 AF, 30 NSR	AF: 66.5 (12.2) YO, NSR: 67.7 (8) YO	25 s	Wrist-type monitor	Outpatient-- checkpoint	3 measurements for the left and right wrist based on irregular pulse peak (IPP) and irregular heartbeat (IHB)	Crafted threshold-based rules	Patient-level performance by IPP 15%: Sen = 0.970; Spe = 1; PPV = 1; NPV = 0.970
Bashar *et al* (2019a)	UMass database 37, Chon Lab database 9	UMass database: 10 AF and 27 non-AF; Chon Lab database: 9 healthy males	—	30 s	Wrist band	Outpatient-- checkpoint	Root mean square of successive differences (RMSSD) and sample entropy (SampEn) from the pulse intervals	Weighted average of two features and threshold-based rule	Sen = 0.982, Spe = 0.974 Acc = 0.975
Bashar *et al* (2019b)	20	8 AF, 12 non-AF	—	30 s	Wristwatch	Outpatient-- checkpoint	Root mean square of successive differences (RMSSD) and sample entropy (SampEn) from the pulse intervals	Weighted average of two features and threshold-based rule	Sen = 0.962, Spe = 0.974 Acc = 0.971
Han *et al* (2019)	16	Patients: 11 NSR and 3 with PAC/PVC, 2 with basal heart rate AF and 3 with fast heart rate AF	63–88 YO	30 s	Smartwatch	Outpatient-- checkpoint	Not an Afib detetion study but the HR estimation study using ppg	—	—
Sološenko *et al* (2019)	34	Clinical testing database with 15 AF and 19 non-AF, plus two simulated developmental and testing databases	AF: 72.9 (8.9) YO, Non-AF 67.5 (10) YO	30 s	PPG simulator	Simulation	PP or RR interval	Threshold based detector using Heaviside step function to calculate sample-entropy like index	Poor SQ dataset: Sen = 0.72, Spe = 0.997;High SQ dataset: Sen = 0.972, Spe = 0. 996.
Han *et al* (2020)	37	All patients have cardiac arrhythmia	50–91 YO	30 s	Wristwatch	Outpatient-- continuous measurement	This is for PAC/PVC detector for AF patients or NSR subject, not for detecting AF	—	—
Inui *et al* (2020)	40	Patients scheduled for cardiac surgery	70.9 (11.1) YO	1 min	Smartwatch and wrist band	Outpatient-- continuous measurement	This is for using ppg for pulse rate estimation in AF as compared to ECG	—	—
Estrella-Gallego *et al* (2020)	9	4 AF, 9 Non- AF	35–80 YO	30 s	Smartphone	Outpatient-- continuous measurement	PPG signals with Offset removed and EWMA filer applied for smoothening	—	—
Väliaho *et al* (2021a)	359	169 AF, 190 NSR	AF: 72.2(14.3), NSR: 57.9 (18.8)	1 min	Wrist band	Inpatient-- checkpoint	The five pulse inverval-based variables were: mean PIN, root-mean-square values of successive differences (RMSSD), AF Evidence (AFE), Coefficient of Sample Entropy (COSEn) and turning point ratio (TPR); Four features based on pulse amplitude were: mean AMP, RMSSD, Sample Entropy (SampEn) and TPR. one autocorrelation feature.	Linear logistic regression	Sen = 0.964 Spe = 0.963 AUC = 0.993
Avram *et al* (2021)	204	32 Non-AF, 159 paroxysmal AF, 16 with persistent AF	62.61 (11.6) YO	5 min	Smartwatch	Outpatient-- continuous measurement	IBI features: the dispersion of the Poincare plot, the standard deviation and the modified Shannon entropy	Logistic regression model	Sen = 0.878 (95% confidence interval [CI] 0.836–0.910) Spe = 0.974 (95% CI 97.10%– 97.70%)
Chorin *et al* (2021)	18	6 AF, 4 DM, 8 HTN, 3 Brugada syndrome, 5 DFT after ICD implant	59.4 ( 21.3) YO	1 min	Cardiac sense smartwatch	Outpatient-- continuous measurement	RR and GG intervals of PPG and ECG	Threshold based defibrillation	
Chang *et al* (2022)	200	112 AF, 88 non-AF	66.1 ( 12.6) YO	5 min	Garmin smartwatch	Outpatient-- continuous measurement	Standard deviation of normal-to-normal intervals and root mean square of successive RR interval	An undisclosed heart rate classifier	Performance based on 5 min segments: Sen = 0.971, Spe = 0.868PPV of AF detection = 0.897
Han *et al* (2022)	35	23 NSR, 5 PAC/PVC, 5 Basal AF, 5 AF with RVR	50–91 YO	30 s	Smartwatch	Outpatient-- continuous measurement	Root mean square of successive differences (RMSSD) and sample entropy (SampEn) from the pulse intervals	Weighted average of two features and threshold-based rule	Not reported. AF detection is a part of the procedure for estimating HR.
Väliaho *et al* (2021b)	173	76 AF, 97 NSR	AF: 77.1(9.7), NSR: 67.3 (15.8)	1 min	Wrist band	Outpatient-- continuous measurement	See Väliaho *et al* (2021b)	Linear logistic regression	30 min time-frame performance: Sen = 0.947, F1 = 0.954
Nonoguchi *et al* (2022)	286	163 with high AF risk, 123 with known AF	66 (12) YO for the high-risk group, 67 (12) YO for AF group	30 min	Wristwatch-type continuous pulse wave monitor	Outpatient-- continuous measurement	Features based on pulse period (PP) values: CV, degree of variation and KS, Kolmogorov–Smirnov difference.	A rule-based algorithm using CV and KS	Patient-level performance: Sen = 0.980 Spe = 0.906 PPV = 0.694 NPV = 0. 995. Interval level performance: Sen = 0.869, Spe = 0.988, PPV = 0.896, and NPV = 0.985

**Abbreviations:** YO—Year Old, s—second, A—atrial fibrillation, NSR—normal sinus rhythm, AFL—atrial flutter, SD—standard deviation, PAC—premature atrial contraction, PVC—premature ventricular contraction, Sen—sensitivity, Spe—specificity, Ac—accuracy, PPV—positive predictive value, NPV—negative predictive value, AUC—area under the receiver characteristic curve, CI— confident interval, DFT—defibrillation threshold, ICD—implantable cardioverter-defibrillator.

**Table 3.** Studies on photoplethysmography based AF detection using ML approaches.

Author (year) [Reference]	Number of patients	Dataset features	Age of population Mean (SD)	Length PPG segments	Measurement device	Acquisition conitions	Input data	Methodology	Performance results for rhythms detection
Yang *et al* (2019)	11	Patients referred to hospital in AF state	63 (12) YO	5, 10, 15, 20 s	Customized wrist-type device	Inpatient	Statistical measures of Wavelet transform coefficients (mean, median, standard deviation, variance, Shannon entropy, energy, contrast, inverse different moment, homogeneity)	Support Vector Machines with polynomial and radial-basis function kernels	Sen = 0.701; Spe = 0.886; Acc = 0.804
Neha *et al* (2019)	15	13 PPG records for training and 2 PPG sample for testing (MIMIC II)	—	24 s	Finger pulse from bedside monitors	Inpatient	Time series features: crest to crest intervals, trough to trough intervals; heart rate.	Artificial neural network (ANN), support vector machine (SVM), Logistic regression, decision trees and Random Forest	Sen = 0.980; Acc = 0.977
Fallet *et al* (2019)	17	All patients referred for catheter ablation of cardiac arrhythmia, 415 VA, 1370 samples of AF and 381 NSR	57 (13 ) YO	10 s	Wrist-type device	Inpatient-- continuous measurement	PPG-wave features and RR time series features	Bagging decision trees	AF versus NSR: Sen = 0.997; Spe = 0.924; Acc = 0.981; PPV = 0.979; NPV = 0.989; F1 = 0.990. AF versus (SR&VA): Sen = 0.962; Spe = 0.928; Acc = 0.950; PPV = 0.959; NPV = 0.934; F1 = 0.960
Guo *et al* (2019)	224	424 suspected AF, 227 confirmed AF	55 to 32 YO	45 s	Wrist-type device	—	Peak-to-peak intervals of ppg for uniform SR, the variance, entropy derived from the peak-to-peak intervals were fluctuating for AF episodes	Threshold based ANN	Sen = 0.93; Spe = 0.84, PPV = 0.85
Zhang *et al* (2019)	375	20 AF, 140 NSR, 47 Hypertension, 23 diabetes, 14 artery disease, 24 current smoking and 32 drinking	Mean age 53 YO	45 s	Wrist-type device	Inpatient-- continuous measurement	Peak to Peak intervals of PPG, Kolmogorov-Smirnov test for normality of continous variables, Normal distributions presenested as Mean (SD), Mann- Whitney Test values for categorical values	Boosting Algorithm	Sen = 0.955; Spe = 0.991; PPV = 0.931; NPV = 1; Kappa = 0.960.
Buś *et al* (2020)	32	8 NSR recordings (total length of 240 min), 24 AF recordings (total length of 120 min); 253 AF samples; 381 NSR samples	—	32 consecutive inter-beat interval (IBI)	Finger pulse wave acquisition system Portapres 2 (FNS, Holland)	—	Mean IBI; standard deviation of IBI; SDSD (standard deviation of the successive differences between IBI);pSD50 (percentage of successive differences between IBI greater than 50 ms)	K Nearest Neighbors (KNN); Support Vector Machine with linear kernel (Linear SVM); Support Vector Machine with radial basis function kernel (RBF SVM); Decision Tree (DT); Naive Bayes (NB).	Best performance: RBF-SVM. Sen, Spe and Acc = over 0.975 (specific performance unavailable due to graphic presentation); F1 = 0.985
Corino *et al* (2020)	200 simulated PPG signals	100 AF, 100 NSR	—	20, 30, 40, 50, 100, 150, 200, 250 and 300 beats	PPG simulator based on phenomenological model	—	Variability analysis of IBI time series; Irregularity of IBI	Linear SVM	Signal length (20 ∼ 300 beats): Sen = 0.881 ∼ 0.991; Spe = 0.940 ∼ 1; Acc = 0.913 ∼ 0.995.
Eerikainen *et al* (2020)	40	276 h of AF, 116 h of atrial flutter (AFL), and 472 h of other rhythms (NSR, and sinus rhythm accompanied by premature atrial or ventrical beats)	Mean age in training set: 66 YO in AF, 63 YO in AFL and 69 YO in Other; Mean age in test set: 76 YO, 70 YO and 72 YO	30 s	Wrist-type data logging device equipped with the Philips Cardio and Motion Monitoring Module	Outpatient-- continuous measurement	IBI features; PPG waveform features and Accelerometer features	Random Forest	AF versus AFL versus Other: Sen = 0.976/0.845/0.981; Spe = 0.982/0.997/0.928; Acc = 0.981/0.964/0.956.
Mol *et al* (2020)	149	PPG recordings are obtained during NSR; AF: 108 records; NSR: 108 records.	69 (9) YO	3 30 s segments	Smartphone	Inpatient	Several rhythm and signal features, such as heart rate variability parameters, peak amplitude, and other signal characteristics	SVM	AF versus NSR: Sen = 0.963; Spe = 0.935; Acc = 0.949
Millán *et al* (2020)	Not mentioned	828 NSR signals and 828 AF signals from five open Physionet datasets	—	—	Finger pulse from bedside monitors	Inpatient-- continuous measurement	IBI time series features, Time–frequency domain features, and Frequency domain features	XGBoost	AF versus NSR: Sen = 0.984; Spe = 0.995; Acc = 0.990
Aydemir *et al* (2020)	7	subject’s signals acquired in squat, stepper and resting phase	20 to 52 YO	3 s window-length PPG	Wrist bracelet	_	Mean, standard deviation, autoregressive model parameter, values of the real part and standard deviation, values of the imaginary part	K-nearest Neighbor, Naïve Bayes, and Decision Tree	Acc = 0.930, CA rate = 0.890
Guo *et al* (2021a)	604	Individuals at high risk for AF.	More than 18 YO	48 sec	Huawei smart device and Holter ECG	Outpatient-- continuous measurement	Heart rate features, Heart rate variability features, Customized AF detection model output probability and Mathematical features	XGBoost	AF versus NSR: Sen = 0.821; Spe = 0.974; Acc = 0.935; PPV = 0.914; F1 = 0.865; AUC = 0.971
Xie *et al* (2021)	21	Healthy participants	—	10 sec	Wearables on forearm	Outpatient-- checkpoint	Wavelet transform based features	SVM	AF versus NSR: Acc = 0.983
Hiraoka *et al* (2022)	80	Patients scheduled for cardiovascular surgery	Mean (SD) 65.8 YO (13.4) after excluding one patient	10 min	Apple watch	Inpatient- continuous measurement	Median value of the mean and SD of PPG pulse rate	Gradient Boosting Decision Tree	AF versus Other: Sen = 0.909; Spe = 0.838
Liao *et al* (2022)	116	76 patients with paroxysmal AF, 40 patients with persistent AF	59.6 (11.4) YO	10, 25, 40, and 80 heartbeats	Wrist-worn smartwatch	Outpatient-- continuous measurement	PPI SD, RMSSD, Shannon entropy (SE10, SE100, and SE1000), rolling SD3, RMSSD3, and MaxFFTSD3 for AF discrimination	Random Forest	AF versus NSR: Sen = 0.941; Spe = 0.934; Acc = 0.937; PPV = 0.930; and NPV = 0.939
Jeanningros *et al* (2022)	42	42 patients refferred for catheter ablation	—	30 s	Wrist bracelet	Outpatient-- continuous measurement	IBI time series features, Frequency domain features, and Pulse wave analysis (PWA) features	Ridge regression, random forest, K-Nearest Neighbors and SVM	AF versus non-AF versus NSR: average Sen = 0.734; Spe = 0.879; Acc = 0.840; PPV = 0.645; NPV = 0.841

**Abbreviations:** YO—Year Old, s—second, AF—atrial fibrillation, NSR—normal sinus rhythm, AFL—atrial flutter, SD—standard deviation, PAC—premature atrial contraction, PVC—premature ventricular contraction, Sen—sensitivity, Spe—specificity, Acc—accuracy, PPV—positive predictive value, NPV—negative predictive value, AUC—area under the receiver characteristic curve, CI—confident interval, DFT—defibrillation threshold, ICD—implantable cardioverter-defibrillator, IBI—inter-beat interval.

**Table 4.** Studies on photoplethysmography based AF detection using DL approaches.

Author (year) [Reference]	Number of patients	Dataset features	Age of population Mean (SD)	Length PPG segments	Measurement device	Acquisition conditions	Input data	Methodology	Performance results for rhythms detection
Shen *et al* (2019)	29+53	13 with persistent AF, 2 with NSR, and 14 with changed rhythm, additional 53 healthy free-living subjects	—	30 s	Samsung wrist-wearable device	Outpatient-- continuous measurement	PPG segment	1D ResNeXt	AUC = 0.950
Rezaei Yousefi *et al* (2019)	30	15 with AF, 15 with NSR	Mean 71.5 YO	30 consecutive PPG pulses	Wrist-worn PPG monitor	Inpatient	IBI features	Deep NN	All data: Sen = 0.936 ± 0.216, Spe = 0. 992 ± 0.180, AUC = 0.996, After quality assessment: Sen = 99.2± 1.3 Spe = 0.995 ± 0.640, AUC = 0.997
Zaen *et al* (2019)	105	84 from Long- Term AF Database from PhysioNet, 21 from Lausanne University Hospital (CHUV)	—	30 s	Tri-axis accelerometer	Outpatient-- continuous measurement	Consecutive IBIs	RNN	Without outlier rejection: Acc = 0.929 Sen = 0.980 Spe = 0.912 F1 = 0.875 With outlier rejection: Acc = 0.986 Sen = 1 Spe = 0.978 F1 = 0.981
Kwon *et al* (2019)	75	57 persistent AF, 18 long-standing persistent AF	Mean 63 YO	30 s	Pulse oximeter	Outpatient-- checkpoint	PPG segment	1D CNN	Sen = 0.993 Spe = 0.959 Acc = 0.976 PPV = 0.960 NPV = 0.993 AUC = 0.998
Torres-Soto and Ashley (2020)	163	107 for cardioversion (CV) group, 41 for exercise stress test (EST) group, and 15 for ambulatory (AM) group	CV: 68 YO EST: 56 YO AM: 67 YO	25 s	Did not specify	Outpatient-- continuous measurement	PPG segment	Autoencoder+1D CNN	Sen = 0.980 Spe = 0.99 F1 = 0.960 FPR = 0.01 FNR = 0.02
Selder *et al* (2020)	60	AF was identi-fied in 6 (10%) subjects, of which 4 were previously undiagnosed	70 (17) YO	60 s	Wrist band	Outpatient-- continuous measurement	PPG segment for quality assessment, 31 features such as RR intervals for AF detection	LSTM for QA, and, Tree based classifier for AF detection	Sen = 1, Spe = 0.960, ACC = 0.970, PPV = 0.750, NPV = 1
Aschbacher *et al* (2020)	51 + 13	40 for algorithms training, 11 for algorithms testing/51 patients were enrolled during cardioversion, additional 13 individual subjects during sleep	63.6 (11.3) YO	Roughly 30 s	Wrist-worn fitness tracker	Inpatient	Model1: RMSSD and RR interval Model 2: 35 consecutive heartbeat Model3: Raw PPG segment	Model1: Logistic regression Model2: LSTM Model 3: DCNN	Model 1: Sen = 0.741 Spe = 0.584 AUC = 0.717 PPV = 0.808 NPV = 0.488 Model2: Sen = 0.810 Spe = 0.921 AUC = 0.954 PPV = 0.960 NPV = 0.671 Model3: Sen = 0.985 Spe = 0.880 AUC = 0.983 PPV = 0.951 NPV = 0.962
Genzoni *et al* (2020)	37	All patients are for catheter ablation procedures and wear an optical heart rate monitor device	—	30 s	Wrist-worn device	Outpatient-- continuous measurement/ inpatient	Consecutive IBIs	GRU	Sen = 1 Spe = 0.966 Acc = 0.979
Chen *et al* (2020)	401	All patients had a stable heart rhythm	>18 YO	71 s	Wrist band	Inpatient and outpatient-- checkpoint	PPG segment	SEResNet	Sen = 0.950 Sep = 0.990 Acc = 0.976 PPV = 0.986 NPV = 0.970
Kwon *et al* (2020)	100	81 for Persistent AF, 19 for long-standing persistent AF	≥20 YO	30 s	Ring-type wearable device	Outpatient-- checkpoint	PPG segment	1D CNN	Sen = 0.990 Spe = 0.943 Acc = 0.969 PPV = 0.956 NPV = 0.987 AUC = 0.993
Aschbacher *et al* (2020)	51	All patients with persistent AF/Patients undergoing electrical cardioversion were sedated and remained supine during the study	63.6 (11.3) YO	10 s	Smartwatc	Outpatient-- continuous measurement	PPG segment	LSTM/CNN	LSTML 0.954 Sen = 0.810 Spe = 0.921 DCNN Sen = 0.985 Spe = 0.880 AUC = 0.983
(Cheng *et al* 2020)	MIMIC-III waveform database: 30000 patients, IEEE dataset: 59 children and 35 adults	60 sick subjects from MIMIC-III, 42 patients from IEEE TBME and 15 h of PPG from synthetic dataset	Children: 0.8–16.5 YO, Adults: 26.2–75.6 YO	10 s	ICU monitor and pulse oximeter	Inpatient and outpatient--continuous measurement	time–frequency chromatograph	CNN-LSTM	Sen = 0.980 Spe = 0.981 Acc = 0.982 AUC = 0.996
Ramesh *et al* (2021)	37	10 with AF, 27 non-AF	—	30 s	Simband	Outpatient-- continuous measurement	Time domain features	CNN	Sen = 0.946±0.02 Spe = 0.952±0.07 Acc = 0.951±0.03F1 = 0.893±0.02 AUC = 0.949 ±0.03
Zhang *et al* (2021a)	53	38 for NSR, 5 for persistent AF and 10 for paroxysmal AF	66.3 (11.8) YO	30 s	Smartwatch	Outpatient-- continuous measurement	PPG segment	multi-view convolutional neural network	Ave of Acc = 0.916 Spe = 0.930 Sen = 0.908
Das *et al* (2022)	175	108 with AF, 67 non-AF	—	25 s	Wrist-worn wearable device	Outpatient-- continuous measurement	PPG segment	Bayesian deep neural network	Without uncertainty threshold: Sen = 0.722 Spe = 0.720 Precision 0.627 F1 = 0.671 AUC = 0.793, Without threshold: Sen = 0.728 Spe = 0.892 Precision 0.783 F1 = 0.754 AUC = 0.858
Ding *et al* (2022)	139	126 for UCLA medical center, 13 for UCSF Neuro ICU	18–95 YO for UCLA medical center, 19–91 YO forUCSF Neuro ICU	30 s	Pulse oximeter	Inpatient-- continuous measurement	PPG segment	ResNet	Sen = 0.928 Sep = 0.988 Acc = 0.961 PPV = 0.985 NPV = 0.943
Sabbadini *et al* (2022)	4158/88	56 from MIMIC database (13 AF), 32 from UQVSD database (2 AF)	—	10 s	PPG device (did not find specified device name?)	Outpatient-- continuous measurement	Root-mean-square (RMS) and the mean of Skewness and Kurtosis	Deep NN	F1 = 0.920, Precision 0.890, Recall 0.950
Nguyen *et al* (2022)	40	18 with NSR, 15 with AF, and 7 with PAC/PVC	—	30 s	PPG sensor patch measured on the wrist	Outpatient-- checkpoint	poincare plot	2D CNN	Sen = 0.968 Spe = 0.989 Acc = 0.981
Liu *et al* (2022)	228	Patients all have arrythmia	52.3 (11.3) YO	10 s	Fingertip PPG sensor	Outpatient-- continuous measurement	PPG segment	1D CNN	AF Spe = 0.934 Acc = 0.944 PPV = 0.890 NPV = 0.940
Neha *et al* (2022)	670 PPG signals/23	400 normal, 90 PVC, 90 tachycardia, and 90 atrial flutters	—	8 s	ICU monitor	Inpatient	Dynamic time warping based features	Deep NN	Sen = 0.970 Spe = 0.970 Acc = 0.960 F1 = 0.960 precision = 0.960
Ding *et al* (2022)	28539 patients, UCSF HER dataset, UCLA dataset, Sim band Dataset, Stanford Dataset	Female AF 2304, Male AF 3473, Female cohort 13203, Male cohort 15330, NSR, PVCs	22 to 65 YO	30 s	Fingerprint, Wearable device	—	PPG segment	Autoencoders + ResNet	AUC = 0.960
Kwon *et al* (2022)	35	All patients underwent successful electrical cardioversion for AF	Mean 58.9 YO	10 s	Smart wring	Outpatient-- continuous measurement	PPG segment	Not specify	AUROC 0.995 Sen = 0.987 Spe = 0.978 FPR = 0.02 FNR = 0.01

Abbreviations: YO—Year old, s—second, AF—atrial fibrillation, NSR—normal sinus rhythm, AFL—atrial flutter, SD—standard deviation, PAC—premature atrial contraction, PVC—premature ventricular contraction, Sen—sensitivity, Spe—specificity, Acc—accuracy, PPV—positive predictive value, NPV—negative predictive value, AUC—area under the receiver characteristic curve, CI—confident interval, DFT—defibrillation threshold, ICD—implantable cardioverter-defibrillator, IBI—inter-beat interval.

When referring to the measurement devices, we classified them into several categories, namely smartwatch, wrist band, fingertip sensor, smart ring, armband, and smartphone. This categorization is based on the implicit location for PPG sensing and the primary utility of the device. For instance, smartwatches and wristbands measure PPG signals at the wrist, while fingertip sensors, smart rings, and armbands measure PPG signals at the fingertip, proximal phalange (i.e. the base of the finger), and various locations within the arm or forearm, respectively. It is important to note that while both smartwatches and wristbands integrate reflective-type PPG sensors at the wrist in all studies, we distinguished between them based on their primary function. Smartwatches, such as the Apple Watch and Samsung Simband, are designed for general-purpose utilization and may include features like a screen and notification management utilities. On the other hand, wristbands, such as the Empatica E4, are screen-less devices primarily intended for monitoring physiological signals. Additionally, studies using smartphones typically perform PPG measurements at the finger using reflective-type PPG sensors, with the camera and flashlight serving as the photosensitive and photoemitter components, respectively.

For studies in which PPG signals were experimentally acquired the vast majority used a reflective-type PPG sensor, which includes form factors such as the smartwatch, wrist band and armband. For studies using PPG signals collected through ‘fingertip sensors’, the working mode (i.e. reflective versus transmissive mode) was not disclosed. Regarding the wavelength of the PPG sensors, this information was not disclosed in more than half of the studies (approximately 57.6%). Moreover, approximately 30.5% and 13.6% of the studies used one or more devices using green and red/infra-red, respectively, making the former wavelength the most common one among studies specifying the device’s wavelength. More information can be consulted in table B1 in appendix B.

### 4.1. Updates on PPG-based AF detection using statistical analysis approaches

A compilation of studies for PPG-based AF detection employing statistical analysis approaches is summarized in table 2. In the interest of maintaining uniformity and enabling systematic evaluation of the advancement in this field in recent years, our study deliberately replicates the table format of tables 1–3 from Pereira *et al* (2020) in our tables 2–4. The table provides an overview of these studies in chronological order, including patient cohorts, data characteristics, employed features and methods, care settings (inpatient versus outpatient), and the resultant performance outcomes. It shows that the statistical analysis approach mainly relies on threshold-based rules on the selected set of features for AF detection. Under this umbrella, the most frequently employed features for AF detection include the RR interval from the ECG and the inter-beat interval (IBI) from PPG (Kabutoya *et al* 2019, Sološenko *et al* 2019, Väliaho *et al* 2019, Väliaho *et al* 2021b, Chang *et al* 2022, Han *et al* 2022). Additionally, the root mean square of successive differences (RMSSD) and sample entropy (SampEn) are also among the most utilized features (Eerikäinen *et al* 2019, Han *et al* 2019, Bashar *et al* 2019b, Avram *et al* 2021, Nonoguchi *et al* 2022). Consequently, the extracted features undergo analysis in terms of their histograms, both with and without the presence of AF and other cardiac rhythms. This analysis assists in determining optimal thresholds that effectively differentiate various rhythmic classes. Once these thresholds are established, they can be applied to the same features extracted from PPG signals.

Furthermore, the utilization of identical feature sets with alternative statistical approaches, such as logistic regression, enhances the versatility and comprehensiveness of AF detection studies. By applying logistic regression, researchers can establish a mathematical model that estimates the probability of AF presence based on the input features. The logistic function, also known as the sigmoid function, is employed to transform the output into a range between 0 and 1. This transformed probability serves as an indicator of the likelihood of AF compared to non-AF cases. The advantage of logistic regression lies in its ability to provide a quantitative measure of the probability, allowing for a nuanced understanding of the classification outcome. Also, as reported in table 2, studies incorporating larger patient cohorts intend to utilize logistic regression (Eerikäinen *et al* 2019, Avram *et al* 2021, Han *et al* 2022) rather than rule-based models. This observation aligns with the trends identified in a previous review study (Pereira *et al* 2020), further reinforcing the preference for logistic regression in cases involving a higher number of patients.

As compared to the previous review, we observe a rising number of studies using the statistical analysis approach (4.25 studies/year between 2019 and 2022 versus 2 studies/year between 2013 ∼ 2019), which aligns with the rising number of all-type AF detection studies in recent years. It can be observed that more studies focus on outpatient populations, which might be attributed to the rapid advancement of wearable technology in recent years.

### 4.2. Updates on PPG-based AF detection using machine learning approaches

Table 3 presents a chronological summary of AF detection studies based on machine learning approaches in the last four years. Machine learning has demonstrated promising results in the detection of AF in low-sample settings. The application of ML techniques requires domain expertise for feature engineering to extract features that effectively capture the comprehensive characteristics of PPG waveforms and enable the discrimination of different classes. Commonly extracted features include morphological descriptors, time domain statistics, statistic measurements in the frequency domain, nonlinear measures, wavelet-based measures, and cross-correlation measures.

Of different machine learning algorithms, Tree-based algorithms, such as decision trees, random forest, and extreme gradient boosting (XGBoost) (Chen and Guestrin 2016), are the most popular choices and are collectively employed in 12 out of the 18 studies employing machine learning for AF detection. Random Forests have demonstrated strong performance in AF detection tasks using PPG. This ensemble learning algorithm combines multiple decision trees to create a robust classification model. By aggregating the predictions of individual trees, Random Forests can reduce overfitting, handle complex feature interactions, and provide accurate AF detection results. The versatility, interpretability, and resilience to noisy data make Random Forests a popular choice in PPG-AF detection research. XGBoost is a boosting algorithm that combines gradient boosting with decision trees to achieve high predictive accuracy in PPG-AF detection. XGBoost sequentially builds an ensemble of weak models, iteratively improving its performance by minimizing a loss function. It can effectively handle complex feature interactions and capture subtle patterns in PPG signals, leading to improved AF classification results and better detection performance compared to individual decision trees.

The second most popular (used in 8 out of 18 studies) machine learning classifier for AF detection is support vector machines (SVM) (Cortes and Vapnik 1995), due to their ability to handle high-dimensional feature spaces. SVM separates PPG signal data into different classes by identifying an optimal hyperplane that maximizes the margin between the classes. By mapping PPG signals into a higher-dimensional space, SVM can capture complex relationships and find effective decision boundaries for accurate AF classification. There are also other classifiers adopted in the studies such as K-Nearest neighbors (KNN) and artificial neural networks (ANN) but are not widely adopted as the above two classifiers.

Compared to the previous review, we observe a sharp increase in the adoption of machine learning for AF detection using PPG (5 studies/year between 2019 and 2022 versus 1.5 studies/year between 2016 and 2019).

### 4.3. Updates on PPG-based AF detection using deep learning approaches

Deep learning has emerged as a powerful approach for detecting AF in PPG signals, as reported in table 4. Unlike traditional ML methods, DL models can learn comprehensive feature representations through an end-to-end learning fashion, eliminating the need for complex feature engineering. This is achieved by learning from a large amount of training samples to train deep neural networks, which consist of interconnected layers of computational nodes.

As shown in table 4, studies using DL approaches can be divided into two main categories. The first category (employed in 14 out of 24 studies) is a family of convolutional neural networks (CNN). CNN is commonly applied in computer vision tasks, but they have also been successfully adapted for PPG-AF detection CNNs utilize convolutional layers to automatically extract relevant features from the PPG signal data (Shen *et al* 2019). These convolutional layers apply numerous filters across the signal, allowing the network to capture local patterns and identify important discriminative features associated with AF. By stacking multiple layers, CNNs can learn increasingly complex representations of the PPG signals, enhancing the accuracy of AF detection. Residual network (ResNet) (He *et al  2016*), a specific type of CNN, addresses the challenge of training deep neural networks by utilizing skip connections. These connections allow the network to bypass layers and pass information directly to subsequent layers, mitigating the vanishing gradient problem. In the context of PPG-AF detection, ResNet architectures enable the training of deeper networks with improved performance and ease of optimization. By incorporating residual connections, ResNet models can capture fine-grained details and long-range dependencies in PPG signals, leading to enhanced AF detection capabilities. The second category is a family of sequential DL models, of which long short-term memory (LSTM) (Hochreiter and Schmidhuber 1997), is a popular choice (employed in 4 out of 24 studies). LSTM is a recurrent neural network architecture commonly used in PPG-AF detection due to its ability to effectively capture temporal dependencies in sequential data. In the context of PPG signals, LSTM models can analyze the sequential nature of the data, considering the temporal order of the signal samples. This allows LSTM to capture long-term patterns and dynamic changes in the PPG signals, which are crucial for accurate AF detection.

To effectively train DL models, a substantial amount of labeled training data is typically required. However, in biomedical applications, the availability of labeled data is often limited. Transfer learning is a potential solution to this challenge, wherein a pre-trained DL model is fine-tuned for a specific task. The number of layers and the complexity of fine-tuning depend on the particular application. For example, in one study, a pre-trained CNN model designed for ECG analysis was fine-tuned to detect AF from PPG segments using a small set of labeled data. Another promising technique is data augmentation to generate artificial samples to boost the number of samples for training the DL models and increasing the generalizability of model performance.

DL is the fastest growing approach of all three approaches for PPG-AF detection. We observe an average of 6 studies employing DL per year between 2019 and 2022, as compared to 3.5 studies/year between 2018 and 2019.

## 5. Discussion

While the performance metrics reported in tables 2–4 suggest the promising potential of PPG for AF detection, several challenges remain. In this section, we will delve into these issues, offering insights drawn from our comprehensive analysis of the reviewed studies. Key concerns to be discussed include PPG signal quality, label accuracy, and the impact of concurrent arrhythmias. Studies that have considered these issues are summarized in table 5. Furthermore, we extend our discussions to encompass additional considerations pertaining to PPG-based AF detection. These include algorithmic factors such as performance metrics, data sources, computational efficiency, domain shifts, as well as model explainability and equity.

**Table 5.** Challenging factors considered in the studies.

Factors	Studies		Capacity
Signal Quality	STAT	(Eerikäinen *et al* 2019, Han *et al* 2019, Kabutoya *et al* 2019, Sološenko *et al* 2019, Bashar *et al* 2019b, Han *et al* 2020, Väliaho *et al* 2021b, Chang *et al* 2022, Han *et al* 2022)	11/17
	ML	(Fallet *et al* 2019, Neha *et al* 2019, Eerikainen *et al* 2020, Mol *et al* 2020, Guo *et al* 2021a, Jeanningros *et al* 2022, Liao *et al* 2022, Zhu *et al* 2022, Neha *et al* 2023)	9/18
	DL	(Kwon *et al* 2019, Rezaei Yousefi *et al* 2019, Zaen *et al* 2019, Chen *et al* 2020, Kwon *et al* 2020, Selder *et al* 2020, Torres-Soto and Ashley 2020, Zhang *et al* 2021b, Das *et al* 2022, Liu *et al* 2022, Neha *et al* 2022, Nguyen *et al* 2022)	12/22
Label noise	STAT	(Väliaho *et al* 2019, Väliaho *et al* 2021b, Chang *et al* 2022)	4/17
	ML	(Fallet *et al* 2019, Hiraoka *et al* 2022, Liao *et al* 2022, Zhu *et al* 2022)	4/18
	DL	(Kwon *et al* 2019, Aschbacher *et al* 2020, Kwon *et al* 2020, Kwon *et al* 2022, Liu *et al* 2022, Nguyen *et al* 2022)	6/22
Concurrent arrhythmias	STAT	(Eerikäinen *et al* 2019, Bashar *et al* 2019b, Han *et al* 2019, 2020, 2022)	5/17
	ML	(Eerikainen *et al* 2020, Liao *et al* 2022)	2/18
	DL	(Kwon *et al* 2019, Genzoni *et al* 2020, Ding *et al* 2022, Liu *et al* 2022)	4/22

### 5.1. PPG signal quality

PPG signal quality remains a considerable challenge, which is widely acknowledged within the scientific community. A multitude of complicating factors can compromise the PPG signal quality, including motion artifacts, skin tone variations, sensor pressure variations, respiratory cycles, and ambient light interference, only to name a few. The challenge of noise in PPG signal is particularly acute when it comes to the continuous acquisition of PPG, which is crucial for long-term monitoring of AF risk.

As reported in table 5, most of the reviewed studies take signal quality into consideration, with 54% of the reviewed studies implementing measures to exclude PPG signals of poor quality. For example, in Han *et al* (2020), the authors presented a noise artifact detection algorithm designed for detecting noise artifacts. Out of a total of 2728 30 s PPG strips, only 314 strips were deemed suitable for further analysis after applying the algorithm. Similarly, in Torres-Soto and Ashley (2020), the authors proposed a multi-tasking framework that incorporated both signal quality assessment and AF detection tasks. Only PPG signals of excellent quality were retained for the purpose of AF detection. This practice, however, harbors potential issues that warrant deeper consideration. Firstly, by systematically discarding vast swaths of signal data considered of inferior quality, the earliest possible detection of AF is inevitably delayed, creating a potentially significant time lag in diagnosis. Secondly, this approach harbors a statistical dilemma; the discarded PPG-AF signals could be construed as false positives within the context of the overall analysis. However, such instances are typically overlooked when calculating the positively predicted value or false positive rate, thereby potentially inflating the model’s reported performance. Consequently, the reliance on selective data exclusion as a signal quality control strategy may inadvertently compromise the validity of the study’s outcomes and the efficacy of predictive models developed therefrom.

We propose a nuanced perspective on PPG signal quality assessment rather than adhering to the dichotomous approach of designating signals as merely black or white (Charlton *et al* 2023). Instead, we suggest the computation of a signal quality index (SQI) as a continuous metric (Guo *et al* 2021b). This calculation would be based on the proportion of motion artifacts present within individual PPG segments, thus providing a more precise estimate of signal quality. Subsequently, an appropriate threshold could be ascertained to filter out PPG signals devoid of meaningful information. Alternatively, one can integrate the signal quality information as part of the model input that controls the uncertainty level of the model output. These approaches would strike the balance of salvaging PPG signals with suboptimal quality for disrupt-less monitoring and model performance.

### 5.2. Label noise

The issue of label noise in annotated datasets presents another significant challenge in the application of PPG for AF detection. Accurate and consistent labeling of datasets is crucial for the development and validation of reliable detection algorithms (Song *et al* 2022). To achieve this, it usually involves more than two clinical domain experts to cross-check the agreement of annotations, and a reconciliation strategy needs to be in place in the event of disagreement. However, many studies often fall short in this aspect due to the labor-intensive task and an insufficient number of cardiologists available to annotate the datasets. Across the reviewed studies, only 9 out of the 57 studies (Kwon *et al* 2019, Väliaho *et al* 2019, Väliaho *et al* 2021b, Chang *et al* 2022, Liao *et al* 2022, Liu *et al* 2022, Nguyen *et al* 2022, Zhu *et al* 2022) employed the expertise of at least two cardiologists for annotation, as reported in table 5. This scarcity of expert annotators can result in imprecise and incomplete labeling of AF events, leading to label noise, which in turn, may undermine the performance of supervised learning algorithms.

Furthermore, the absence of standardized guidelines to address disagreements among annotators exacerbates this issue. In the event of conflicting annotations, the lack of a clear protocol or consensus mechanism can lead to inconsistencies in the dataset. This variability not only confounds the training of predictive models but also hampers the reproducibility of research findings. Consequently, establishing robust procedures for data annotation, which involve recruiting sufficient expert annotators and defining clear rules for resolving disagreements, is paramount. Addressing these issues would significantly enhance the quality of the annotated PPG datasets, thereby facilitating more reliable and accurate AF detection.

In addition to the shortage of expert involvement, the field faces another substantial challenge: the absence of clear clinical guidelines for annotating AF events using PPG data. Unlike ECG, which has well-established guidelines for AF event labeling, PPG operates in a far less standardized environment. This lack of formalized guidance further exacerbates the risk of label noise, compromising both algorithmic performance and clinical reliability. Given these constraints, it becomes imperative to consider multimodal signal inputs when annotating data. Incorporating ECG or other established modalities alongside PPG can provide a more robust framework for annotation, thereby improving the quality of labeled data.

Also, table 6 provides an overview of the methodologies used in obtaining annotated PPG data across the three study categories. Among the 57 studies, a significant majority (47 studies, representing approximately 80% of the studies) relied on annotated ECG data as the primary reference for validating PPG data during the classification phase, emerging as the predominant approach for generating ground-truth data. Furthermore, four studies used direct PPG data labeling, while three studies adopted mixed annotation techniques, which included simulated data (i.e. PPG data was generated based on acquired and annotated ECG signals). Notably, in three instances, the specific methodology for ground-truth generation was not explicitly outlined.

**Table 6.** Summary of the data annotation methods across the three study categories.

Ground-truth	Studies		Capacity
Simultaneously acquired ECG signals	STAT	(Eerikäinen *et al* 2019, Han *et al* 2019, Kabutoya *et al* 2019, Sološenko *et al* 2019, Väliaho *et al* 2019, Bashar *et al* 2019b, Estrella-Gallego *et al* 2020, Han *et al* 2020, Inui *et al* 2020, Avram *et al* 2021, Chorin *et al* 2021, Väliaho *et al* 2021b, Chang *et al* 2022, Han *et al* 2022, Nonoguchi *et al* 2022)	17/17
	ML	(Fallet *et al* 2019, Guo *et al* 2019, Yang *et al* 2019, Buś *et al* 2020, Corino *et al* 2020, Eerikainen *et al* 2020, Millán *et al* 2020, Mol *et al* 2020, Guo *et al* 2021a, Hiraoka *et al* 2022, Jeanningros *et al* 2022, Liao *et al* 2022, Zhu *et al* 2022)	13/18
	DL	(Kwon *et al* 2019, Rezaei Yousefi *et al* 2019, Shen *et al* 2019, Zaen *et al* 2019, Aschbacher *et al* 2020, Chen *et al* 2020, Genzoni *et al* 2020, Kwon *et al* 2020, Selder *et al* 2020, Torres-Soto and Ashley 2020, Ramesh *et al* 2021, Zhang *et al* 2021a, Kwon *et al* 2022, Liu *et al* 2022, Neha *et al* 2022, Nguyen *et al* 2022, Ding *et al* 2023)	17/22
Labeled PPG signals	STAT	—	0/17
	ML	(Neha *et al* 2019, 2023)	2/18
	DL	(Neha *et al* 2019, Das *et al* 2022)	2/22
Mixed annotation methods or simulated data	STAT	—	0/17
	ML	(Aydemir *et al* 2020)	1/18
	DL	(Cheng *et al* 2020, Ding *et al* 2022)	2/22
Unknown	STAT	—	0/17
	ML	(Zhang *et al* 2019, Xie *et al* 2021)	2/18
	DL	(Sabbadini *et al* 2022)	1/22

### 5.3. Concurrent arrhythmias

The detection accuracy of AF through PPG can be significantly influenced by the presence of other arrhythmias, notably premature ventricular contractions (PVC), premature atrial contractions (PAC), and atrial flutter (AFL). All of these introduce irregularities into the heart rhythm that can mimic the rhythm irregularities seen in AF, potentially leading to false-positive detections. PVCs and PACs are characterized by early heartbeats originating from the ventricles and atria, respectively (Han *et al* 2020). These early beats can disrupt the regular rhythm of the heart, resulting in PPG signal patterns that may resemble those associated with AF. Whereas in AFL, the rhythm is typically more organized and less erratic than AF, presenting a sawtooth-like pattern in ECG tracings which does not typically manifest in PPG data (Eerikainen *et al* 2020). This organized rhythm may not exhibit the characteristic variability and irregularity that PPG-based AF detection models are designed to identify. Consequently, a PPG-based AF detection model might mistakenly classify these as AF events, thereby reducing the specificity of the model. Furthermore, the simultaneous presence of AF and other arrhythmias in the same patient adds another layer of complexity to the problem. This co-existence can modify the PPG signal’s morphology in ways that differ from the signals of patients with AF or PVC/PAC alone, making it more difficult to accurately identify the presence of AF.

It is noteworthy that several studies considered the presence of arrhythmias other than AF, as shown in table 5. For instance, in the study by Eerikainen *et al* (2020), Liao *et al* (2022), the differentiation of PVC and PAC from AF using PPG signals was explored. The results of this investigation demonstrated successful differentiation between PVC/PAC and AF based on PPG signal characteristics. Despite limited research on PPG-based detection of atrial flutter (AFL), Eerikäinen *et al* have shown that PPG can differentiate among AF, AFL, and other rhythms. They employed a Random Forest classifier that utilizes a combination of inter-pulse interval features and PPG waveform characteristics, achieving high sensitivity and specificity (Eerikainen *et al* 2020). These findings suggest that PPG-based analysis holds promise for distinguishing various types of arrhythmias beyond AF. Thus, when developing and evaluating PPG-based AF detection models, it is critical to account for the potential influence of other arrhythmias. Robust algorithms should be designed to discriminate between AF and these other rhythm disturbances to maintain high detection accuracy, reinforcing the necessity of comprehensive, diverse, and well-annotated training datasets in the development of these predictive models.

### 5.4. Quantitative metrics for algorithm performance evaluation

The studies reviewed in this work always use conventional performance metrics, such as the area under the receiver operational characteristics curve (AUROC), accuracy, sensitivity, specificity, and F1 Score. However, it is crucial to acknowledge that relying solely on these conventional metrics may be insufficient, particularly within the context of continuous health monitoring scenarios (Butkuviene *et al* 2021). The landscape of continuous health monitoring, facilitated through wearable devices, unfolds as a dynamic and perpetually evolving terrain of data. Within this context, the intrinsic nature of a continuous data stream introduces complexities that transcend the conventional boundaries of traditional evaluation metrics. In scenarios wherein health-related parameters undergo ceaseless scrutiny, the spectrum of fluctuations, subtleties, and overarching trends assumes paramount significance. Conventional metrics, by design, tend to compartmentalize performance assessment within discrete segments, potentially missing the panoramic context that is intrinsic to continuous health monitoring. This paradigm invites us to reflect upon the necessity of embracing evaluation methodologies that are attuned to the temporal dynamics, such as assessing the frequency of AF occurrence that reflects AF burden, the duration of AF episodes, the nuances of variation, and the holistic import of trends. For instance, incorporating equivalent standards to the ANSI/AAMI EC57:2012 standard (which is used for ECG) (American Association of Medical Instrumentation 2020) into algorithm evaluation frameworks for PPG-based AF detection could provide guidance for assessing the clinical significance in continuous monitoring scenarios.

### 5.5. Domain shift problem

PPG signals, despite their utility in non-invasive physiological monitoring, present certain complexities linked to the site of acquisition and inter-patient variability. It has been observed that PPG signals sourced from distinct anatomical sites yield diverse morphological patterns (Fleischhauer *et al* 2023). This is primarily due to the different vascular structures, skin thickness, and other physiological attributes specific to these sites. Such morphological variations can pose significant challenges in interpreting these signals and developing universally applicable models, as the distribution of signal characteristics is inherently contingent on the site of collection.

Moreover, inter-patient variability further compounds this issue by introducing additional variations in the data distribution. These variations stem from a wide array of factors, including demographic attributes (such as age and sex), physiological characteristics (including skin pigmentation and body mass index [BMI]), and medical conditions unique to individual patients (Clifton *et al* 2007). For instance, an older patient might exhibit a different PPG signal morphology due to increased arterial stiffness, while individuals with darker skin might present a different signal-to-noise ratio owing to higher melanin content that can observe more light than lighter skin.

These site-specific and inter-patient differences can induce what is referred to as a ‘domain shift’ problem in machine learning (Wang and Deng 2018, Radha *et al* 2021). Here, a model that is trained on data from a specific group (for example, PPG signals from a certain body site or a particular patient group) may not generalize the model performance when it is applied to a different group. Therefore, while harnessing PPG signals for health monitoring and disease prediction, it is paramount to consider these variations and devise strategies to address the domain shift problem for reliable and generalized model performance.

### 5.6. Lack of large-scale labeled dataset

In concert with the label noise issue discussed in section 5.2, there exists a challenge of a paucity of large-scale, annotated datasets. To develop robust and reliable algorithms for AF detection, especially when deep learning models are employed, it requires extensive, labeled datasets. These ideal datasets should encompass a broad range of patient demographic groups, diverse health conditions, and various physiological states to ensure generalizable findings. Furthermore, they should contain precise annotations of the AF events in the PPG signal to facilitate effective supervised learning.

Emerging research is increasingly focused on addressing this issue by generating synthetic PPG signals through various data augmentation techniques. These range from traditional computational models that simulate physiologic PPG patterns (e.g. PPGSynth) (Tang *et al* 2020) to advanced generative models such as generative adversarial networks (GANs) (Goodfellow *et al* 2020, Ding *et al* 2023), variational autoencoders (VAEs) (Kingma and Welling 2013), and diffusion models. However, the extent to which these synthesized signals contribute to improved learning outcomes remains an open question. Recent research by Cheng *et al* indicates the existence of a ‘performance ceiling’—a limit to the improvements achieved by incorporating synthetic signals (Ding *et al* 2023). This underscores the need for further investigation into more effective algorithms for synthetic signal generation as well as a deeper understanding of this performance ceiling phenomenon.

To sum up, the lack of large, labeled datasets impedes the progress of research in this area, limiting the development and validation of predictive models. It restricts the ability to comprehensively evaluate and compare the performance of different AF detection methods under diverse and challenging conditions. Additionally, it hampers the exploration of more advanced machine learning techniques, which often necessitate large quantities of annotated data to train effectively. Therefore, efforts to collect/generate, share, and consolidate large-scale, well-annotated PPG datasets for AF detection represent a critical step to move the performance needle in this field.

### 5.7. Computational time

With the rapid advancement of graphics processing units (GPUs) and increasing computational power, it is now feasible to train complex, large-scale neural networks that outperform traditional statistical or conventional machine learning methods (Thompson *et al* 2020). However, this complexity presents new challenges, particularly for model inference. The inference process, which involves generating predictions from new data based on trained models, can be computationally demanding. This poses significant obstacles for wearable technologies that rely on edge computing, as these calculations can quickly deplete battery life, thereby undermining the feasibility of continuous monitoring (Chen and Ran 2019). Alternative solutions include offloading computational tasks to more powerful, tethered smartphones or to cloud-based platforms. Yet, both alternatives require robust and fast data streaming infrastructures.

Research efforts to address these challenges are bifurcated. On one hand, there is a burgeoning focus on ‘tiny ML,’ which aims to optimize neural network architectures for efficient edge computing without sacrificing performance. On the other hand, advancements in hardware and battery technology are driving the development of more powerful sensing techniques that enhance the capacity for long-term monitoring. Consequently, tackling these computational challenges necessitates orchestrated efforts from both research directions. It also underscores the imperative to keep computational requirements at the forefront when developing PPG-based AF detection algorithms.

### 5.8. Explainability

Explainability in the context of PPG AF detection algorithms is a critical aspect that determines how well we understand the decision-making process of these algorithms. This is particularly important in healthcare, where the decisions made by these algorithms can have significant implications for patient care. Statistical methods are often considered naturally explainable because they rely on well-understood mathematical principles and procedures. For example, a linear regression model, which lies in the intersection between statistical methods and machine learning, makes predictions based on a weighted sum of input features. The weights (or coefficients) assigned to each feature provide a direct measure of the feature’s importance in the prediction, making it relatively straightforward to interpret the model’s decisions. Machine learning methods, on the other hand, often involve more complex computations and may not be as directly interpretable as statistical methods. However, techniques have been developed to calculate feature importance, which can provide a certain level of explainability. For instance, in Yang *et al* (2019), the Fisher score method was employed to calculate the importance of features. The Fisher score is a statistical measure that evaluates the discriminative power of individual features in a classification task. By utilizing this method, the study aimed to assess the relevance and significance of different features in the context of atrial fibrillation detection. Similarly, in Jeanningros *et al* (2022), each feature was input into the classifier separately, enabling the generation of a ranked list based on its impact on the overall classification performance through this sensitivity analysis.

Deep learning models, on the other hand, are often referred to as ‘black boxes,’ which make predictions based on intricate, high-dimensional mappings that are difficult to comprehend for humans. While they may achieve high predictive accuracy, it’s often challenging to understand what features and their interactions the models use to make predictions, and how these features contribute to the final decision. This lack of transparency can be a major drawback in healthcare applications, where it’s desirable to understand the underlying decision logic so as to gain trust from end users, such as clinicians and patients.

Several approaches are being explored to improve the explainability of deep learning models, including attention mechanisms, layer-wise relevance propagation, and model-agnostic methods like local interpretable model-agnostic explanations (LIME) and SHapley Additive exPlanations (SHAP) (Binder *et al* 2016, Ribeiro *et al* 2016, Zhou *et al* 2016, Lundberg and Lee 2017). A good example is Liu *et al* (2022), where authors used the guided gradient-weighted class activation mapping (Grad-CAM) approach to visualize crucial regions within the PPG signals that enabled the model to predict a specific rhythm category. Despite these advances, explainability in deep learning remains an active area of research, particularly in the context of PPG-based AF detection.

### 5.9. Performance bias and model equity

Disparities in both access to and outcomes from utilizing digital health solutions and biotechnologies manifest a variety of identity dimensions, including economic status, social background, ethnicity, and gender (Lanier *et al* 2022). As described by Braveman (2014), health equity means, ‘…striving for the highest possible standard of health for all people and giving special attention to the needs of those at greatest risk of poor health, based on social conditions.’ In the context of PPG-based AF detection, this issue of equity extends across a spectrum of potential causes. It encompasses accessibility issues, particularly for individuals from rural areas or those with disadvantaged socioeconomic statuses, as well as physiological factors like skin tone and obesity, which can influence the reliability of PPG readings (Ajmal *et al* 2021, Fine *et al* 2021). Of the studies reviewed, a mere three explicitly touched upon the issue of performance bias and model equity (Aschbacher *et al* 2020, Avram *et al* 2021, Zhang *et al* 2021b). This oversight underscores the pressing need to heighten awareness and equity considerations within the field. To tackle this challenge, a multidisciplinary approach is necessary, and healthcare providers, engineers, and researchers must proactively develop technologies that consider the needs of vulnerable and underrepresented populations.

## 6. Conclusion

In conclusion, this comprehensive review highlights the growing significance of PPG-based AF detection in addressing a critical clinical challenge. The surge in research efforts, especially in machine learning and deep learning approaches, underscores the potential of PPG technology for continuous and accurate AF monitoring. While machine learning techniques offer versatility and promising results, deep learning models demonstrate remarkable performance by automating feature extraction. Nevertheless, challenges related to signal quality, label accuracy, and concurrent arrhythmias persist, necessitating ongoing research and development. Furthermore, the availability of large-scale labeled datasets, computational efficiency, model explainability, and addressing performance bias and equity issues emerge as crucial considerations in advancing PPG-based AF detection technology. This review underscores the importance of continued collaboration between the medical and artificial intelligence communities to refine and deploy effective solutions for AF detection, ultimately improving patient outcomes in the face of this widespread health concern.

## Data Availability

The data cannot be made publicly available upon publication because no suitable repository exists for hosting data in this field of study. The data that support the findings of this study are available upon reasonable request from the authors.

## Appendix A

**Table A1.** Summary of the train/test data splitting and excluded data due to noisy data and motion artifacts (STAT).

Author (year) [References]	Initial data	Excluded data	Excluded data (%)	Total Acquisition time after exclusion	Train/test data split
Väliaho *et al* (2019)	220 subjects	Initially 220 subjects, with 7 subjects excluded due to inadequate quality of data or inconclusive rhythm	3.2% patients	8.8 h (AF patients), 8.9 h (Sinus Rhythm control)	Unspecified (probably from ECG as GT)
Eerikäinen *et al* (2019)	40 subjects	8 patients (40 min)	20% patients/signal length	5 min x 32 patients = 160 min	leave-one-subject-out cross- validation
Kabutoya *et al* (2019)	59 patients	—	—	150 seconds x (29 AF + 30 SR); total of 1,180 beats	Unspecified (probably from ECG as GT)
Bashar *et al* (2019b)	2394 segments	UMass dataset: 2080 30 s acquisitions (17.3 h)	86.9% of PPG segments	UMass dataset: 314 segments were clean and used (55 AF and 259 non-AF); Chonlab: NSR subjects (9 subjects; 285 segments) and 52 30 s segments were clean/used	Unspecified (probably from ECG as GT)
Han *et al* (2019)	491 segments	428 segments	87.2% of segments (for the proposed motion noise artifact signal-quality criteria)	30 sec segments x 63 total segments (proposed MNA: Motion and Noise Artifacts)	Unspecified (probably from ECG as GT)
Han *et al* (2019)	—	—	—	141 30 s segments are detected as clean data from the 16 patients (11 with SR and 5 with cardiac arythmia)	—
Sološenko *et al* (2019)	—	—	10.8% of signals	316 h for AF and 411 h for non-AF	—
Han *et al* (2020)	2728 30 s segments	314 30 s segments	MNE 88.5%	(all 37 subjects with cardiac arythmia) 2728 x 30 sec for training dataset (2 subjects, one AF and one non-AF) 101 x 30 sec for Samsung Gear S3 Dataset 4 h of PPG data for testing dataset MIMIC III Dataset (2 AF, 5 NSR and 3 PAC/PVC)	Train: 37 subjects; Test: first 2 subjects (1 AF and 1 non-AF) and then 10 subjects with (2 AF, 5 NSR and 3 PAC/PVC)
Inui *et al* (2020)	33 AF events from 40 enrolled subjects	10 AF events due to device-related noises and interruptions	30% of AF events	23 AF events	—

**Table A2.** Summary of the train/test data splitting and excluded data due to noisy data and motion artifacts (STAT continued).

Author (year) [Reference]	Initial data	Excluded data	Excluded data (%)	Total Acquisition time after exclusion	Train/test data split
Estrella-Gallego *et al* (2020)	—	—	—	2 min x 9 subjects = 18 min	—
Väliaho *et al * (2021a)	365 PPG signals/subjects with confirmed rhythm	6 PPG signals/subjects	1.6% of PPG signals/subjects	1 min x 359 subjects	10-fold cross validation
Avram *et al* (2021)	207 participants with collected signals	3 participants excluded	1.4%	A total of 81 944 h of monitoring from the ePatch with simultaneous W-PPG data was recorded and analyzed.	—
Chorin *et al* (2021)	1527 QRS complexes	—	—	1527 QRS complexes	—
Chang *et al* (2022)	24 h × 200 subjects	—		24 h × 200 subjects	Training: 25 subjects with cardiac arythmia
Han *et al* (2022)	—	—		Training: 35 participants, testing: 25 subjects; 271 segments for training; 2112 clean 30 s for testing	Training/testing datasets
Väliaho *et al* (2021b) ( 2021b)	3781 h of PPG data were analyzed,	1667 h of PPG data	44% of PPG data	2114 h (55.9%) of the data were approved by the quality algorithm.	
Nonoguchi *et al* (2022)	40 055 intervals were obtained	13985 intervals exclude (7022 due to insufficient PWM and 6963 due to insufficient telemetry ECG)	34.9% of intervals	163 × 30 min segments (hig risk) with 17 segments of AF and 123 × 30 min (known AF) with 55 segments of AF	ECG

**Table A3.** Summary of the train/test data splitting and excluded data due to noisy data and motion artifacts (ML).

Author (year) [References]	Initial data	Excluded data	Excluded data (%)	Total Acquisition time after exclusion	Train/test data split
Yang *et al* (2019)	13.2 h for NSR and 23.7 h for AF episodes	—	—	13.2 h for NSR and 23.7 h for AF episodes	75% of the time slots for training and 25% for testing.
Neha *et al* (2019)	24 s × 15 subjects (6 min)	—	—	24 s × 15 subjects (6 min)	13 PPG samples (for training) and 2 PPG samples (for testing)
Fallet *et al* (2019)	2166 labeled 10-s epochs from 17 patients	—	—	2166 labeled 10 s epochs from 17 patients	5-fold cross validation
Guo *et al* (2019)	227 individuals, with 186 956 identified AF episodes	11 individuals	4.8%	216 entered the follow-up program	—
Zhang *et al* (2019)				—	—
Buś *et al* (2020)	8 PPG recordings for NSR (240 min) and 24 for AFib (120 min)	None	0%	8 PPG recordings for NSR (240 min) and 24 for AFib (120 min)	—
Corino *et al* (2020)	Simulated PPG signals corresponding to 20, 30, 40, 50, 100, 150, 200, 250 and 300 RR intervals. For each length, 200 signals were generated, 100 in AF and 100 in NSR.	—	—	Simulated PPG signals corresponding to 20, 30, 40, 50, 100, 150, 200, 250 and 300 RR intervals. For each length, 200 signals were generated, 100 in AF and 100 in NSR	train-validation-test split (55% of the data is used as training set, 25% as validation set, 20% as test set
Eerikainen *et al* (2020)	39 subjects, each on a 24 h-acquisition (936 h total)	Train: 368 h; Test: 138.7 h	Train: 53%; Test: 57.8%. Average per subject	Train: 328 h; Test: 101.3 h	75% of the patients to the training set and 25% to the test set
Mol *et al* (2020)	216 × 90 s	Only the second recording attempt was considered for each subject	—	216 × 90 s	—
Millán *et al* (2020)	—	—	—	—	1656 signals for training

**Table A4.** Summary of the train/test data splitting and excluded data due to noisy data and motion artifacts (ML continued).

Author (year) [Reference]	Initial data	Excluded data	Excluded data (%)	Total Acquisition time after exclusion	Train/test data split
Aydemir *et al* (2020)	5 h 30 min	—	—	5h30min	½ train and ½ test
Guo *et al* (2021a)		—	—	469 267 PPG signals (optimization setp); 30 640 PPG signals for AF and 89 359 PPG signals for non-AF (for the testing step)	Randomly divided (3:1)
Xie *et al* (2021)	PPG signals collected form 21 healthy individuals	—	—	—	—
Neha *et al* (2023)	100 8 s PPG signals (800 s)	signals with multiple abnormalities in a frame have been excluded from the study	—	—	70:30 ratio train test
Zhu *et al* (2022)	Total of 106 663 h of collected PPG signals	34 345 h of PPG signals	32.2%	72 317 h of PPG signals	A pre- viously trained model is deployed in real-world setting
Hiraoka *et al* (2022)	Average of 13.3 d of PPG measurements among 80 patients (24 h monitoring)	1 subject excluded from the measurements	—	Average of 13.3 d of PPG measurements among 79 patients (24 h monitoring)	training cohort of 59 patients and a test cohort of 20 patients
Liao *et al* (2022)	—	—	18%	—	five-fold cross-validation
Jeanningros *et al* (2022)	11985 30 s windows (99.9 h)	7838 30 s window (65.3 h)	65.4%	4147 30 s windows (34.5 h)	leave-one-group-out

**Table A5.** Summary of the train/test data splitting and excluded data due to noisy data and motion artifacts (DL).

Author (year) [Reference]	Initial data	Excluded data	Excluded data (%)	Total Acquisition time after exclusion	Train/test data split
Rezaei Yousefi *et al* (2019)	30 subjects	1 subjects	3.3%	22.5 h (estimate) from 29 subjects (1.5 h average PPG acquisition each)	29 subsets were used as the test set and the remaining 28 subsets were put together to form a training set
Zaen *et al* (2019)	physionet dataset: unknow; CHUV dataset: 21 subjects	—	Physionet dataset; unknown; CHUV dataset: 0% (no outlier rejection) or 50% (with outlier rejection)	Physionet dataset: 1719 h CHUV dataset: unknwon	80%/20% split stratified by label; for physionet (only ECG)
Kwon *et al* (2019)	119.2 h	Unknown	unknown	119.2 h	10 × 5-fold cross-validation
Aschbacher *et al* (2020)			—	72 total hours + 91 h	40 sujects to train and 11 to test
Torres-Soto and Ashley (2020)	>500k labeled signals	—	—	Evaluation datasets: Held out test set: 151.7; ambulatory cohort 156.5 h	Train/test from multiple datasets. Train: The model is trained on approximately one million simulated unlabeled physiological signals and fine-tuned on a curated dataset of over 500 K labeled signals from over 100 individuals from 3 different wearable devices.
Selder *et al* (2020)	180 min of PPG signals (1 min per PPG signal) from 60 subjects	43 PPG signals	24%	137 PPG signals/minutes	trained on full data + manually annotated PPG-signals
Genzoni *et al* (2020)	3213 30 s segments (26.8 h) of labeled segments	849 30 s noisy segments (7 h)	26.4%	2364 30 s labeled segments (19.7 h)	Trained on one public db and tested on theirs

**Table A6.** Summary of the train/test data splitting and excluded data due to noisy data and motion artifacts (DL continued).

Author (year) [Reference]	Initial data	Excluded data	Excluded data (%)	Total acquisition time after exclusion	Train/test data split
Chen *et al* (2020)	401 patients	15 patients	3.7%	386 × 3 min (19.3 h)	Training step: 70% of the total data set was randomly selected for the training step, 90% of which was used as the training set, and 10% was used as the cross-validation set; Testing step: 30% of the total data
Kwon *et al* (2020)	108,6 h (13 038 30-s PPG)	—	—	108,6 h (13 038 30 s PPG)	5-fold cross validation repeated 10 times
Aschbacher *et al* (2020)	91 h of PPG recordings	—	—	91 h of PPG recordings	80%/20% train/test
Cheng *et al* (2020)	60 subj x 1 h (MIMIC-III) + 42 subj x 8 min (IEEE TBME) + 15 h (synthetic)	—	—	60 subj x 1 h (MIMIC-III) + 42 subj x 8 min (IEEE TBME) + 15 h (synthetic)	training set, val- idation set, and test set with a 6:2:2 ratio.
Ramesh *et al* (2021)	37 subjects, with 10 having AF	—	—	37 subjects, with 10 having AF	80% was randomly divided for training and validation, and 20% was used as the test set. The Stratified k-fold cross-validation strategy was implemented with *k* = 5
Zhang *et al* (2021a)	27 622 h	None	None	27 622 h	5-fold cross- validation scheme with a random selection
Das *et al* (2022)	872 h of PPG signals in raw dataset	—	None for raw dataset, 97.4% for ‘Excellent quality’ segments	872 h of PPG signals in which the algorithm was tested or 3246 25 s segments (22.5 h) for ‘Excellent quality’ segments	split of 70% as train, 15% as validation, and 15% as test sets (Based on subjects)
Ding *et al* (2022)	—	—	—	No time range. Estimated for testing: UCLA medical center: 1349 h; Stanford dataset 3681 h; Simband dataset 2.9 h	Training: UCSF EHR;Testing: UCLA medical center, Stanford dataset, Simband dataset
Sabbadini *et al* (2022)	4158 windows 10 s (11.5 h)			4158 windows 10 s (11.5 h)	

**Table A7.** Summary of the train/test data splitting and excluded data due to noisy data and motion artifacts (DL continued).

Author (year) [Reference]	Initial data	Excluded data	Excluded data (%)	Total Acquisition time after exclusion	Train/test data split
(Nguyen *et al* 2022)	Pre-train: MIMIC III (1327 h for ECG, and 21 h for PPG), Train (qualified data) 79.5 min;test (qualified) 23 min	—	—	Pre-train: MIMIC III (1327 h for ECG, and 21 h for PPG), Train (qualified data) 79.5 min;	
test (qualified) 23 min	Pre-train (test 70% train 30% both for ECG and PPG in transfer learning) and then main experiment: 80% train 20% test				
(Liu *et al* 2022)	158 355 10 s segments (439.9 h)	30 793 10 s segments (85.5 h)	19.4%	354 h	—
(Neha *et al* 2022)	89 min	—	—	89 min	—
Ding *et al* (2022) (Ding *et al* 2023)	—	—	—	Train: 1467 h; test: 22.4 h	Train: 126 patients UCLA Medical Center Test: UCSF Medical Center
(Kwon *et al* 2022)	Total of 2532 PPG-ECG snapshots were acquired (from 35 participants, average of 9.2 d continuous acquisition)	909 PPG-ECG snapshots	35.9%	1623 PPG-ECG snapshots	—

## Appendix B

**Table B1.** Summary of the color ranges used in PPG sensors. Some studies used wearable devices with more than one color range, which were included in more than one category.

Color/ wavelength	Studies	Capacity	Capacity (%)
Green	(Eerikäinen *et al* 2019, Fallet *et al* 2019, Kabutoya *et al* 2019, Rezaei Yousefi *et al* 2019, Väliaho *et al* 2019, Zaen *et al* 2019, Aydemir *et al* 2020, Chen *et al* 2020, Eerikainen *et al* 2020, Han *et al* 2019, 2020, Zhang *et al* 2021a, Väliaho *et al* 2021b, Hiraoka *et al* 2022, Nguyen *et al* 2022, Ding *et al* 2023, Neha *et al* 2023)	18/57	31.5
Red/IR	(Fallet *et al* 2019, Aydemir *et al* 2020, Selder *et al* 2020; Väliaho *et al* 2019, 2021b, Nguyen *et al* 2022, Ding *et al* 2023)	8/57	14.1
Smartphone flashlight (white)	(Estrella-Gallego *et al* 2020, Mol *et al* 2020)	2/57	3.5
Unspecified	(Guo *et al* 2019, Han *et al* 2019, Kwon *et al* 2019, Neha *et al* 2019, Sološenko *et al* 2019, Yang *et al* 2019, Zhang *et al* 2019, Bashar *et al* 2019b, Aschbacher *et al* 2020, Buś *et al* 2020, Cheng *et al* 2020, Corino *et al* 2020, Genzoni *et al* 2020, Inui *et al* 2020, Kwon *et al* 2020, Millán *et al* 2020, Mol *et al* 2020, Torres-Soto and Ashley 2020, Avram *et al* 2021, Ramesh *et al* 2021, Xie *et al* 2021, Guo *et al* 2021a, Chang *et al* 2022, Das *et al* 2022, Ding *et al* 2022, Han *et al* 2022, Jeanningros *et al* 2022, Liao *et al* 2022, Liu *et al* 2022, Neha *et al* 2022, Nonoguchi *et al* 2022, Sabbadini *et al* 2022, Zhu *et al* 2022)	34/57	59.6
